# Development of an Electrochemical Biosensor Based on Polypyrrole-3-carboxylic Acid/Polypyrrole/Au Nanoparticle Composites for Detection of Dopamine

**DOI:** 10.3390/polym17060754

**Published:** 2025-03-13

**Authors:** Rapiphun Janmanee, Saengrawee Sriwichai

**Affiliations:** 1 Chemistry Program, Faculty of Science and Technology, Pibulsongkram Rajabhat University, Phitsanulok 65000, Thailand; 2 Department of Chemistry, Faculty of Science, Chiang Mai University, Chiang Mai 50200, Thailand; 3 Center of Excellence in Materials Science and Technology, Chiang Mai University, Chiang Mai 50200, Thailand

**Keywords:** polypyrrole, gold nanoparticle, dopamine, electrochemical, biosensor

## Abstract

Dopamine (DA) is a neurotransmitter that works in the brain. It plays several important roles in executive functions, including motor control, memory, mood, motivation, and reward. DA imbalances are associated with diseases in the nervous system such as Parkinson’s disease, schizophrenia, Alzheimer’s disease, and attention deficit hyperactivity disorder (ADHD). Therefore, the development of a biosensor for the detection of precise amounts of DA is of great interest. In this research, polypyrrole-3-carboxylic acid/polypyrrole/gold nanoparticle (PP3C/PPy/AuNPs) composites were developed for the electrochemical detection of DA. Firstly, a PP3C/PPy/AuNPs composite thin film was synthesized by electropolymerization on a fluorine-doped tin oxide (FTO)-coated glass substrate. Subsequently, cyclic voltammetry (CV), scanning electron microscopy (SEM), and differential pulse voltammetry (DPV) were used for the characterization and study of the efficiency of the obtained conducting polymer–gold nanoparticle composite thin film for the detection of DA. The proposed electrochemical sensor showed good sensitivity and selectivity for the detection of DA with a wide detection linear range from 5 to 180 μM (R^2^ = 0.9913). The limit of detection (LOD) and limit of quantitation (LOQ) values were 9.72 nM and 0.032 μM, respectively. Therefore, it can be concluded that the electrochemically fabricated PP3C/PPy/AuNPs composite thin film can be applied as an electrochemical biosensor for the detection of dopamine for the early diagnosis of various neurological disorders in the future.

## 1. Introduction

Dopamine (DA) is an important neurotransmitter in biological systems [1,2]. DA significantly influences various cognitive and physiological processes, including mood regulation [3], motivation [4], endocrine regulation [5], and movement [6]. The normal DA concentration in the nervous and peripheral systems can range from 0.01 to 1000 μM [7,8,9,10]. DA dysfunction causes several neurodegenerative diseases and psychiatric disorders, such as Parkinson’s disease, Huntington’s disease, Alzheimer’s disease, and depression [4,5,10,11]. Consequently, it is essential to accurately and sensitively determine DA levels in biological fluids for both research and clinical applications. Various analytical methods have been utilized for monitoring DA levels including calorimetric methods [12], chromatographic methods [13], spectrophotometric methods [14], and immunoassay [15]. These methods have unique advantages but are greatly limited due to their operational complexity, high costs, time-consuming processes, and tedious sample pre-treatments [16,17,18]. Hence, to address this issue, the development of an electrochemical biosensor for DA determination has been extensively considered.

Currently, electrochemical biosensors using several techniques, including cyclic voltammetry (CV) [19], amperometry [20], differential pulse voltammetry (DPV) [21], or square-wave voltammetry [22], have been actively explored for DA level detection due to their simple operation, cost-effectiveness, rapid response, and high sensitivity and selectivity [23,24,25]. However, in biological matrices, the high concentration of ascorbic acid (AA) and uric acid (UA) is a huge problem in DA detection. The overlapping oxidation peaks of DA, AA, and UA pose challenges to the accurate detection of DA [22,26,27]. Consequently, most researchers are focused on the enhancement of the sensitivity and selectivity of electrochemical biosensors for DA detection in biological samples containing various interferences.

To date, the surface modification of working electrodes with nanomaterials and nanocomposites, including conducting polymers (CPs), noble metal nanomaterials, metal oxide nanomaterials, and carbon-based materials, has been focused on enhancing their selectivity and sensitivity [24,26,28,29]. Among the various materials, CPs have been extensively used in electrochemical biosensors due to their excellent electrical properties, biocompatibility, and environmental stability [30,31,32]. Polypyrrole (PPy) and its derivatives are among the most widely used CPs for electrochemical biosensor fabrications due to their good environmental stability in air and aqueous media, facile synthesis via both chemical and electrochemical methods, and higher electronic conductivity compared to many other CPs [25,33,34,35,36]. Moreover, to improve the performance of PPy-based electrochemical biosensors, the synthesis of composite materials by combining PPy with nanomaterials, such as metal oxides, carbon nanotubes (CNTs), graphene, or metal nanoparticles (NPs), has attracted much interest [1,37,38,39,40,41,42].

Recently, composite nanomaterials based on CPs and NPs of various metals, including Ag, Pt, Pd, and Au, have been increasingly utilized in the fabrication of electrochemical sensors to improve their analytical performance [11,43,44,45,46,47]. Gold nanoparticles (AuNPs) are one of the metal nanoparticles that serve as an advantageous material for PPy-based electrochemical biosensors due to their great conductivity, good electrocatalytic activity, and enhanced electron transfer with a large surface area [25,29]. Therefore, an electrochemical dopamine biosensor was developed using PPy-decorated AuNPs as a composite to enhance the CPs’ properties in this study.

**Scheme 1:**
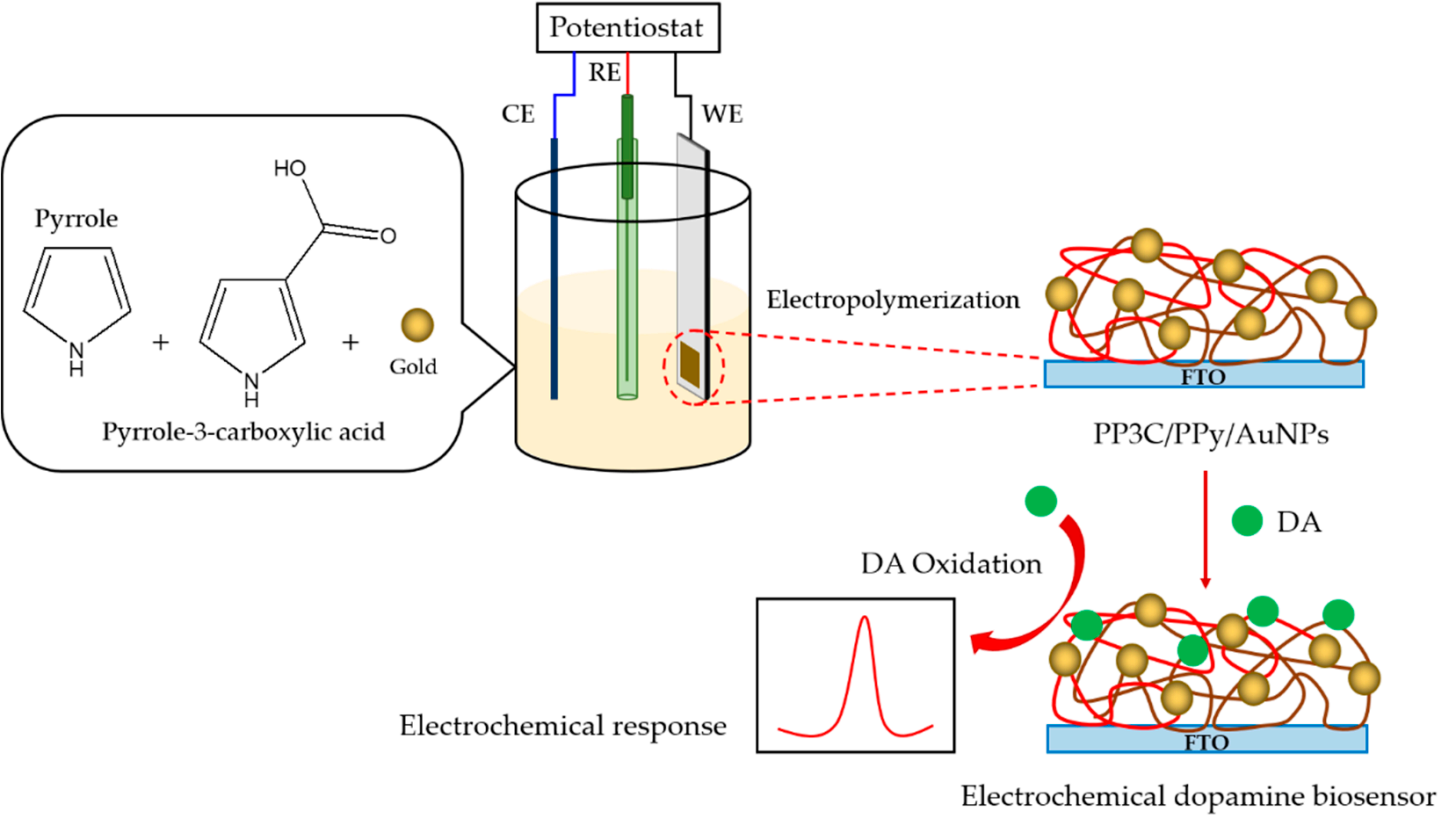
Schematic diagram of the fabrication of PP3C/PPy/AuNPs composites for use as electrochemical DA biosensors.

In this present study, we have attempted to integrate the advantages of nanomaterials based on CPs and NPs, utilizing a composite material composed of polypyrrole-3-carboxylic acid, polypyrrole, and gold nanoparticles (PP3C/PPy/AuNPs) to prepare an electrochemical biosensor for detecting DA with high sensitivity and selectivity. From this interesting perspective, the presented composite of this PP3C/PPy/AuNPs for electrochemical dopamine biosensors has never been reported. First, the PP3C/PPy/AuNPs composite was synthesized by electropolymerization under optimal conditions. The fabrication of the proposed electrochemical DA biosensor based on PP3C/PPy/AuNPs composites is presented in Scheme 1. The fabricated PP3C/PPy/AuNPs composite thin film exhibited excellent electrochemical performance for label-free DA detection using the DPV technique. The developed electrochemical biosensor based on PP3C/PPy/AuNPs demonstrated high selectivity for detecting DA with no interference effects from AA and UA. The proposed PP3C/PPy/AuNPs thin film showed excellent efficiency for application as an electrochemical biosensor for DA detection in real sample analysis, which has the potential to be applied in the future of human health diagnoses.

## 2. Materials and Methods

### 2.1. Chemicals and Materials

Dopamine (DA), gold solution colloidal (18–20 nm), pyrrole (Py), pyrrole-3-carboxylic acid (P3C), ascorbic acid (AA), uric acid (UA), and phosphate-buffered saline (PBS) were acquired from Sigma-Aldrich (Darmstadt, Germany). Potassium chloride (KCl) and sulfuric acid (H_2_SO_4_) were purchased from RCI Labscan (Bangkok, Thailand). Potassium hexacyanoferrate (K_3_Fe(CN)_6_) was purchased from Fisher Scientific (Mumbai, India). Potassium hexacyanoferrate (II) trihydrate (K_4_Fe(CN)_6_) was acquired from Loba Chemie™ (Mumbai, India). Deionized water (DI) was applied for all aqueous solutions. All chemicals were of analytical grade and used as received.

### 2.2. Electropolymerization of PP3C/PPy/AuNPs

The PP3C/PPy/AuNPs composite thin film was fabricated by electropolymerization with the following procedure: Firstly, 10 mM of P3C/Py monomer solution was prepared by dissolving 0.120% *w*/*v* P3C and 0.065% *w*/*v* Py in 0.5 M H_2_SO_4_. Then, 40 µL of 0.0065wt% colloidal solution of gold was added to 10 mM of P3C/Py precursor solution and sonicated for 5 min. The electropolymerization of PP3C/PPy/AuNPs was performed using CV using a standard three-electrode electrochemical cell setup. A fluorine-doped tin oxide (FTO)-coated glass substrate (Sigma-Aldrich, Darmstadt, Germany) was used as a working electrode (area of 0.25 cm^2^). The counter electrode was Pt wire, and the reference electrode was Ag/AgCl (3 M KCl). The potential was scanned from −1.0 to 1.0 V for 5 cycles. The scan rate was set at 20 mV/s. The individual PP3C and PPy were both produced under the same electrochemical polymerization conditions of the PP3C/PPy/AuNPs composite thin film.

### 2.3. Structural Characterizations of PP3C/PPy/AuNPs

The electrochemical characteristics of the fabricated PP3C/PPy/AuNPs in 10.0 mM of freshly prepared K_3_Fe(CN)_6_/K_4_Fe(CN)_6_ (1:1, containing PBS solution, pH 7.4) ([Fe(CN)_6_]^3−/4−^) were monitored using CV between −0.5 to 1.0 V under different scan rates (5–100 mV/s) on a potentiostat Autolab204 (Metrohm Autolab B.V., Utrecht, The Netherlands) with a three-electrode system. The electrochemical cell consisted of the Ag/AgCl reference electrode in 3 M KCl, Pt counter electrode, and FTO working electrode. The NOVA 2.1 software was employed to simulate all the electrochemical data reported in this research. Morphological studies were examined using scanning electron microscopy (SEM, Thermo Fisher Scientific, Apreo S, Waltham, MA, USA). The functional groups of the composite were identified by attenuated total reflectance–Fourier transform infrared spectroscopy (ATR-FTIR, Bruker Tensor 27, Billerica, MA, USA). X-ray photoelectron spectroscopy (XPS, AXIS ultra DLD spectrometer, Manchester, UK) was used to characterize the chemical constituents of the prepared composite. All specimens intended for XPS analysis were prepared on an ITO glass substrate.

### 2.4. Determination of DA

The electrochemical detection of DA based on the obtained PP3C/PPy/AuNPs-modified FTO electrode was monitored using the CV and DPV techniques. The CV measurement for 20 μM DA in PBS solution was recorded at 20 mV/s with a potential between −1.0 and 1.0 V. In addition, DPV measurements were performed to study the sensitivity performance. The potential ranged between −0.2 to 1.0 V with a scan rate of 20 mV/s, while the DA concentration was varied from 5 to 180 μM. The DPV responses were investigated at 20 µM of DA containing 200 µM AA and 200 µM UA as common interferences in the evaluation of selectivity. For stability determination, the biosensor electrodes were tested at two-day intervals for 10 days. Three similar independent electrodes were employed for the detection of 100 μM DA to evaluate reproducibility.

## 3. Results and Discussion

### 3.1. Fabrication of PP3C/PPy/AuNPs

**Figure 1:**
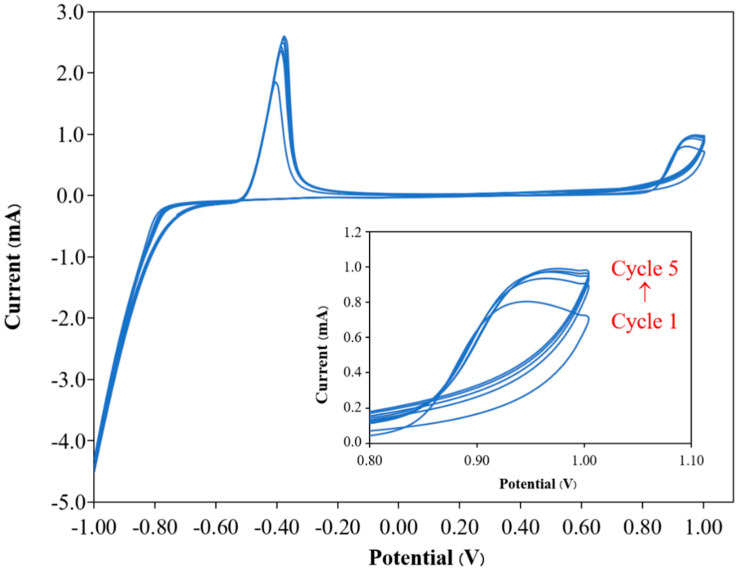
CV responses of the fabricated PP3C/PPy/AuNPs composite thin film.

The electrochemical property of the PP3C/PPy/AuNPs composite thin film was investigated using the CV technique. Figure 1 shows the CV curves of the PP3C/PPy/AuNPs composite thin film. The current in the oxidation onset began at about 0.6 V, and the current during the cathodic scan slightly decreased at about 0.3 V, which is attributed to the beginning of the formation of the copolymer film and the dedoping process of the copolymer films on the FTO electrode, respectively [37]. The peak current appeared at about −0.3 V, corresponding to the presence of gold nanoparticles on the modified working electrode. The CV responses of the PP3C/PPy/AuNPs composite thin film and the PPy electrode are presented in Figure S1. It was found that only the CV response of the PP3C/PPy/AuNPs electrode exhibited notable current peaks at −0.3 V, which were associated with the presence of AuNPs in the PP3C/PPy/AuNPs composite thin film. Moreover, it was found that the oxidation/reduction currents of the PP3C/PPy/AuNPs composite thin film increased with the number of cycles, as shown in the insets of Figure 1. This indicates an increase in the thickness of PP3C/PPy/AuNPs [48]. Furthermore, the detailed structural morphologies and chemical compositions of the PP3C/PPy/AuNPs composite thin film were investigated using SEM, ATR-FTIR, and XPS techniques.

### 3.2. Characterization of PP3C/PPy/AuNPs Composite Thin Film

**Figure 2:**
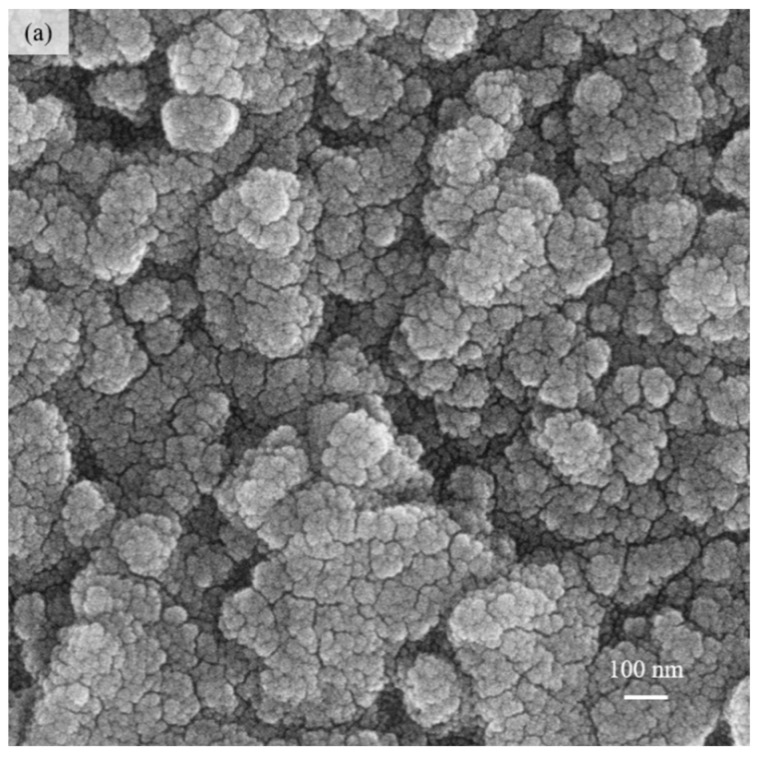

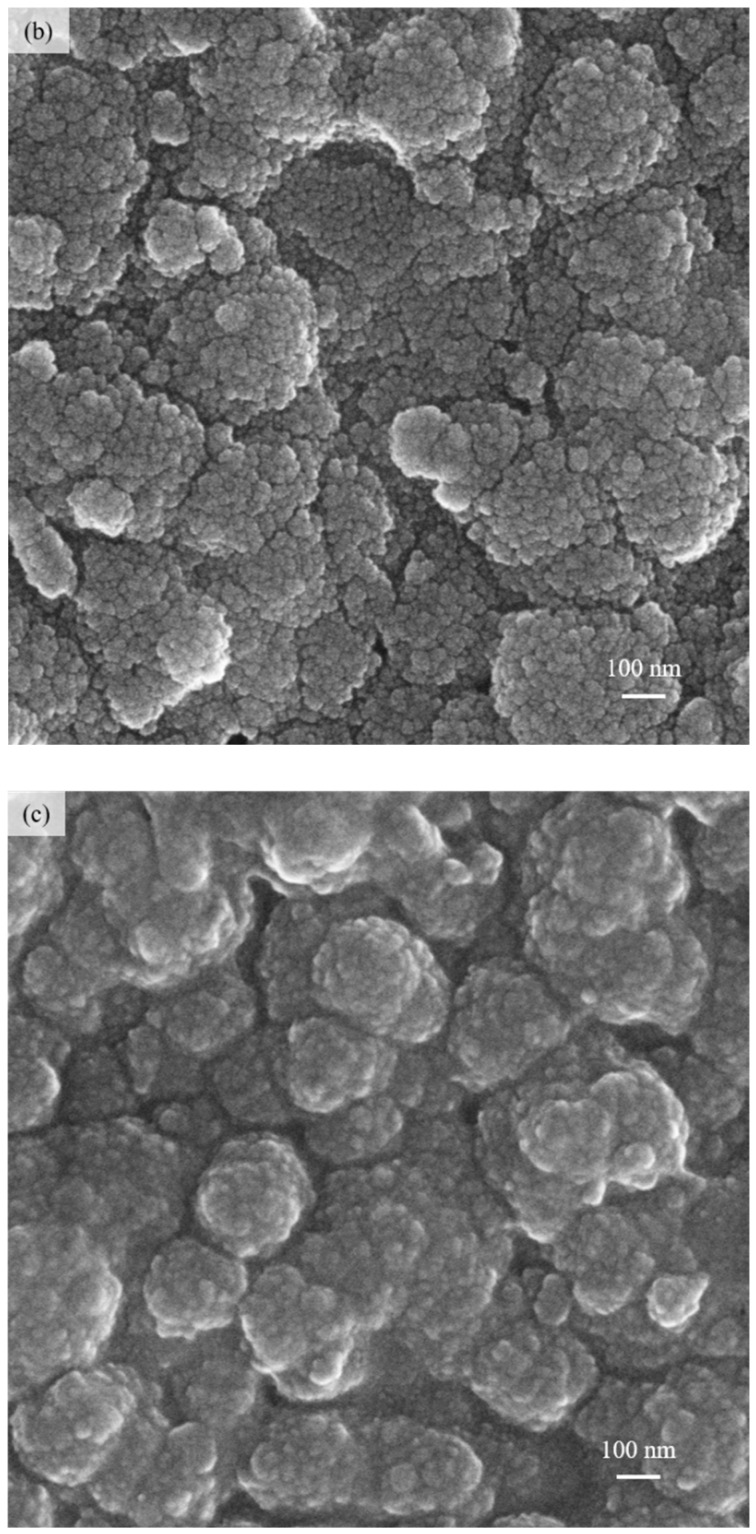
Surface morphology of (**a**) PP3C, (**b**) PPy, and (**c**) PP3C/PPy/AuNPs by SEM analysis.

The SEM was applied to characterize the surface morphological features of the PP3C PPy and PP3C/PPy/AuNPs, as illustrated in Figure 2. The morphology of the PP3C was a spherical morphology with nanoscale structures in random clusters, as seen in Figure 2a. Figure 2b shows the granular morphology of PPy, which is densely packed, with granules smaller than those in PP3C. As for the PP3C/PPy/AuNPs composite, the SEM image in Figure 2c reveals a cauliflower-like morphology of PP3C/PPy/AuNPs, and the granules are larger than those in PP3C and PPy. It suggests a higher reaction area and larger amounts of catalytic sites for electrochemical reactions [1,34,49]. The SEM-EDX spectrum illustrated in Figure S2 clearly demonstrates the presence of AuNPs within the fabricated PP3C/PPy/AuNPs.

**Figure 3:**
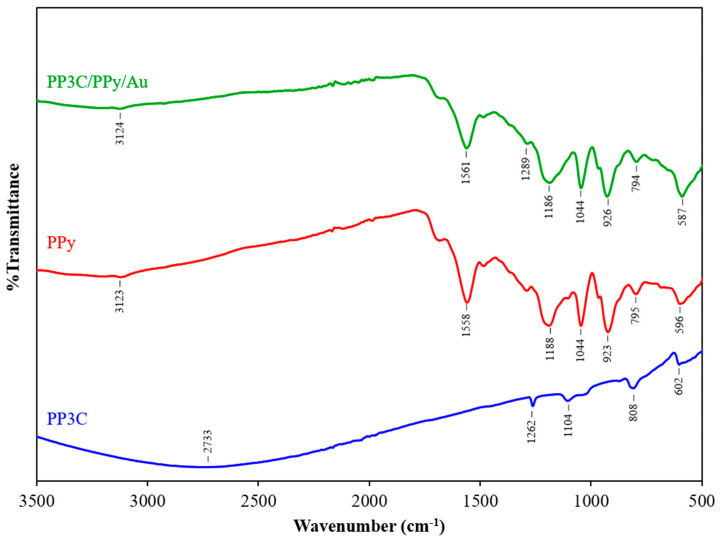
ATR-FTIR spectra of PP3C, PPy, and PP3C/PPy/AuNPs.

The functional groups of the prepared PP3C, PPy, and PP3C/PPy/AuNPs were identified by ATR-FTIR spectroscopy. The ATR-FTIR spectra of PP3C, PPy, and PP3C/PPy/AuNPs in the range of 500–3500 cm^−1^ are presented in Figure 3. In the ATR-FTIR spectra of the PP3C, a very broad band peak at around 2400–3400 cm^−1^ is ascribed to the stretching vibration of O–H and –C=O of the carboxylic acid from P3C. The C–O of the carboxylic acid group showed a peak at 1289 cm^−1^ [50]. The bipolaron band of aromatic rings for PP3C appeared at 1104 cm^−1^ [34]. The C–H out-of-plane and the C–C out-of-plane ring deformation in the PP3C structure showed peaks at 808 cm^−1^ and 602 cm^−1^, respectively [48]. In the ATR-FTIR spectra of PPy, the peak observed at 3123 cm^−1^ is due to the C–H stretching of the pyrrole ring [51]. Peaks appeared at 1558 1188 cm^−1^ and 1044 cm^−1^, indicating the stretching vibrations of C=C and C–N bonds and the C–H in-plane vibration, respectively. The N–H in-plane vibrations are presented at 923 cm^−1^. The bending vibrations of C–H out of plane are represented by the peak at 795 cm^−1^ [51,52]. The peak at 596 cm^−1^ indicates ring deformation [53]. The ATR-FTIR spectrum of the PP3C/PPy/AuNPs composite represents all characteristic peaks of both PP3C and PPy. Moreover, a red shift for PP3C/PPy/AuNPs (1561 cm^−1^) indicates the doping of AuNPs through the PP3C/PPy/AuNPs composite [48]. The results demonstrate that the PP3C/PPy/AuNPs composite could be successfully synthesized through electrochemical polymerization.

**Figure 4:**
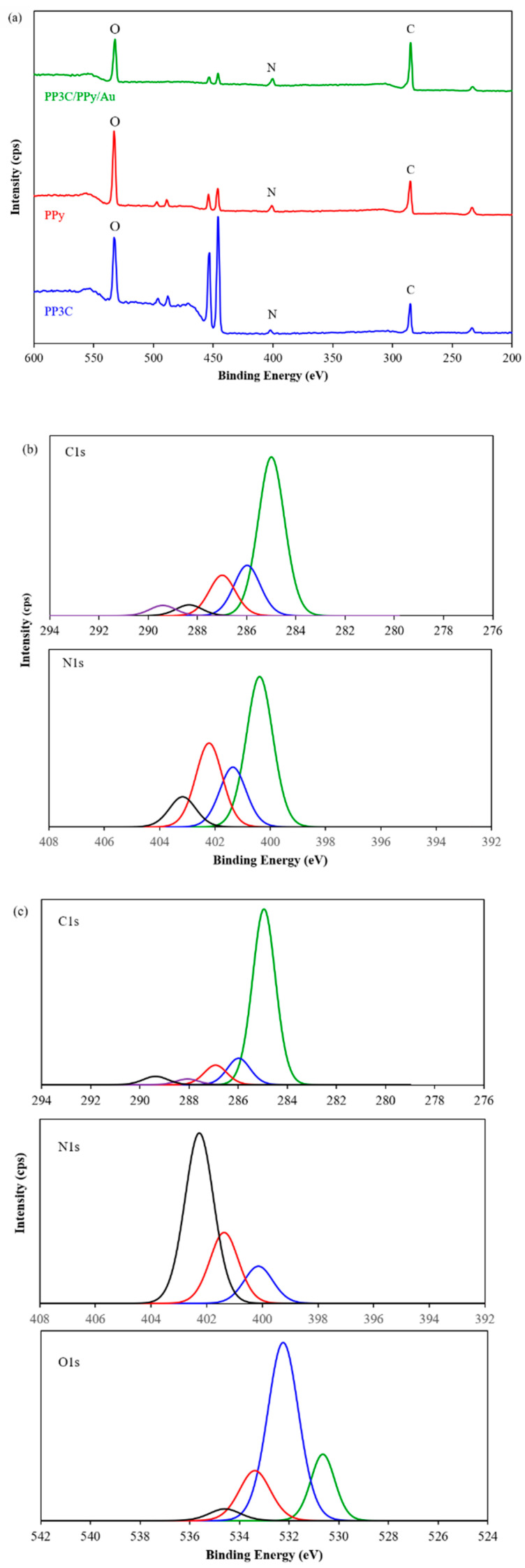

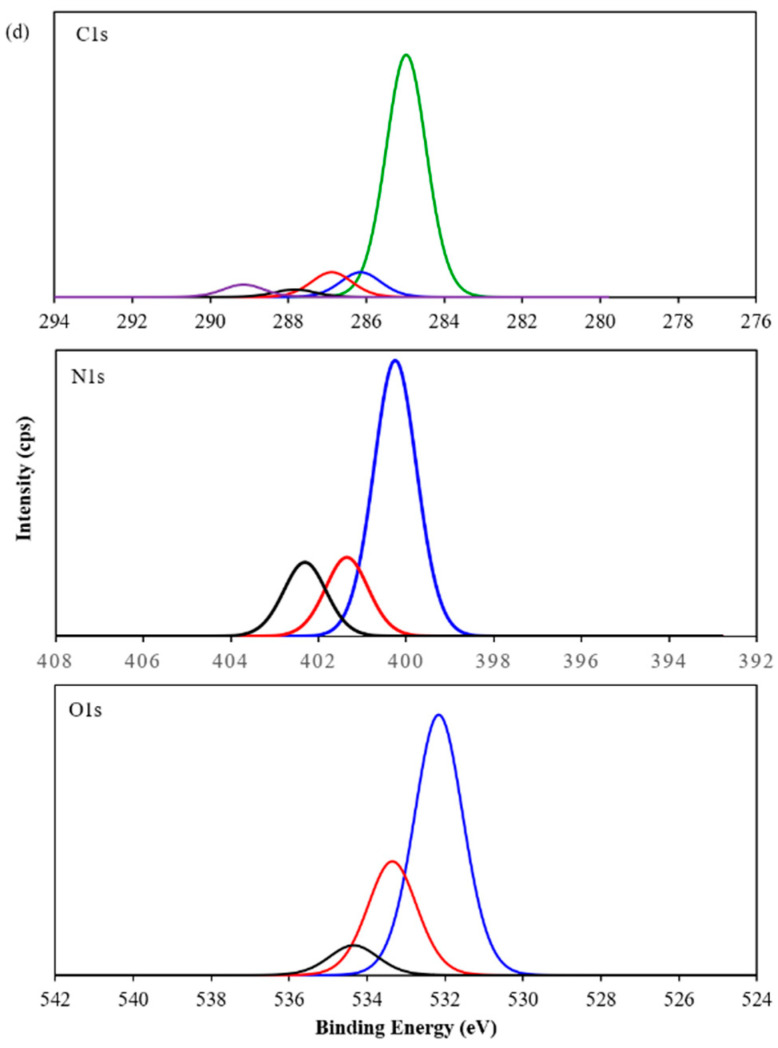
(**a**) Survey scan of PPy, PP3C, and PP3C/PPy/AuNPs and XPS spectra of (**b**) PPy, (**c**) PP3C, and (**d**) PP3C/PPy/AuNPs.

The illustrative XPS data of PP3C, PPy, and PP3C/PPy/AuNPs are displayed in Figure 4. The C1s, N1s, and O1s peaks appeared at a binding energy of about 285, 402 and 533 eV, respectively, as shown in the survey spectra (Figure 4a). The peak is observed within the energy range of 440–460 eV, indicating the presence of the In spectrum associated with the ITO glass substrate. The C1s, N1s, and O1s spectra of PPy, PP3C, and PP3C/PPy/AuNPs are presented in Figure 4b, c, and d, respectively. For PPy, the C1s spectra showed binding energy at 284.9 (green line), 285.9 (blue line), 286.9 (red line), 288.3 (black line), and 289.3 eV (purple line), corresponding to C–C, C–N, C–O, C=O, and π = π* [54,55]. The C1s spectra of PP3C showed binding energy at 284.9 (green line), 285.9 (blue line), 286.9 (red line), 288.1 (purple line), and 289.4 eV (black line). The C1s regions of the XPS spectrum of PP3C/PPy/AuNPs are similarly found at 284.9 (green line), 286.1 (blue line), 286.9 (red line), 287.8 (black line), and 289.2 eV (purple line), respectively. The deconvoluted N1s spectra of PPy can be divided into four component peaks at 400.4 (green line), 401.3 (blue line), 402.2 (red line), and 403.1 eV (black line), corresponding to –NH–, –N^+^–, NH^+^–, and –NH_2_^+^, respectively [7,56]. The N1s spectra of PP3C and PP3C/PPy/AuNPs exhibited the identical –NH–, –N^+^–, and =NH^+^– at 400.2 (blue line), 401.3 (red line), and 402.3 eV (black line), respectively. The O1s spectra of PP3C showed four peaks at 530.6 (green line), 532.2 (blue line), 533.4 (red line), and 534.6 eV (black line), respectively. The peak at 530.6 is due to O–C, 532.2 eV can be associated with the carbonyl (O=C) group, and the 533.6–534.6 eV is due to the carboxyl group (–COOH). All the peaks that appeared in the O1s region of PP3C were also present in the PP3C/PPy/AuNPs composite. The XPS results indicate that the presented PP3C/PPy/AuNPs composite could be successfully formed in this study.

**Figure 5:**
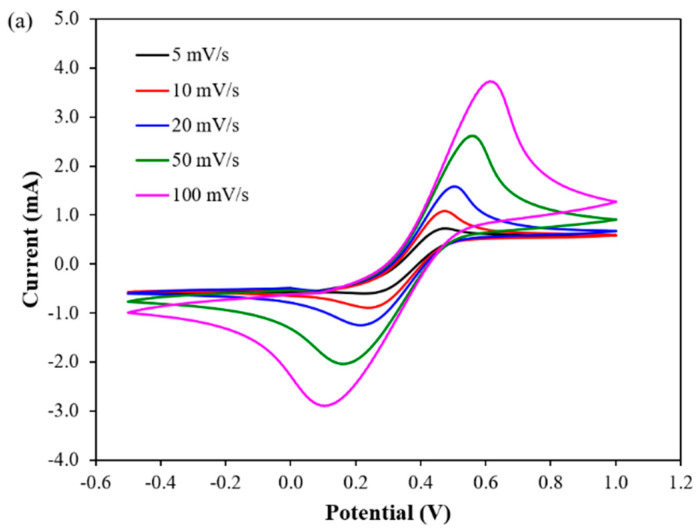

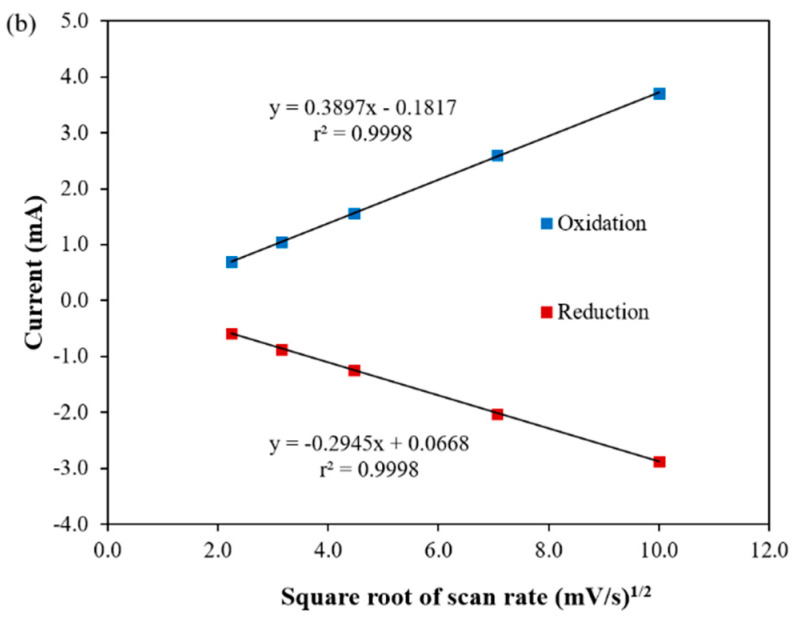
(**a**) CVs and (**b**) plot of peak current of PP3C/PPy/AuNPs at various scan rates.

Furthermore, the electrochemical property of the PP3C/PPy/AuNPs-modified electrode in [Fe(CN)_6_]^3−/4−^ solution was studied. The CV responses of the PP3C/PPy/AuNPs at various scan rates from 5 to 100 mV/s are presented in Figure 5a, and the redox peak current increased with the increase in the scan rate. Figure 5b shows a good linear relationship in the plot of the oxidation and reduction peak current with the square root of scan rates, which indicated the diffusion-controlled redox process. It could be concluded that the PP3C/PPy/AuNPs composite thin film had sufficient electrochemical reactivity for device development [57].

### 3.3. Electrochemical Detection of DA at PP3C/PPy/AuNPs

**Figure 6:**
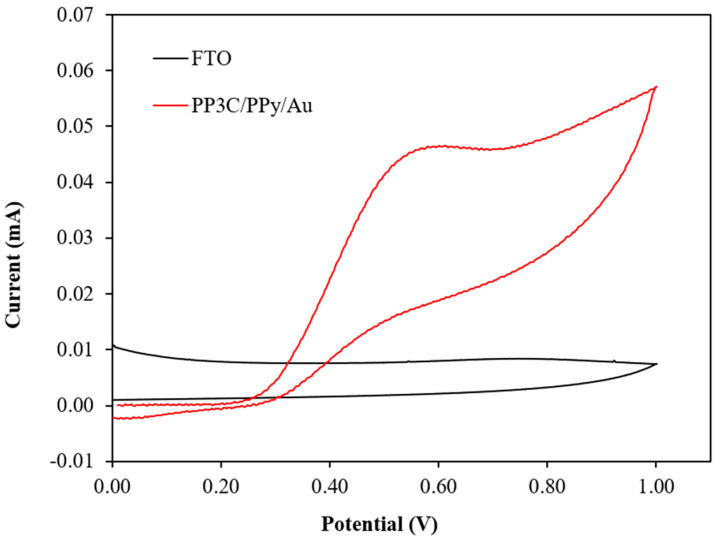
CV of FTO and PP3C/PPy/AuNPs in the presence of 20 µM dopamine.

**Figure 7:**
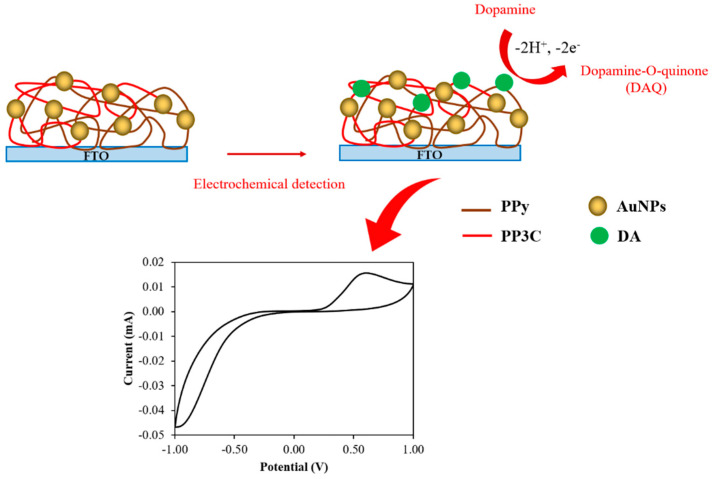
The electrochemical mechanism of PP3C/PPy/AuNPs for the detection of DA.

The electrochemical biosensor based on the obtained PP3C/PPy/AuNPs-modified electrode was compared to the bare FTO electrode for the detection of DA using the CV technique. Figure 6 presents the current responses of the PP3C/PPy/AuNPs composite thin film-modified electrode and the bare FTO electrode to 20 μM DA in PBS solution. The CV response of the bare FTO electrode shows no significant current peaks during the electrochemical processes. On the other hand, the proposed electrochemical DA biosensor demonstrated a high current response. These results are attributed to the interactions between the redox species of dopamine and the electrode. [4]. Figure 7 represents the schematic of the electrochemical process for detecting DA based on the PP3C/PPy/AuNPs composite thin film-modified electrode. When an electrode potential is applied, dopamine is easily oxidized to dopamine-O-quinone (DAQ), leading to the two electrons and protons being transferred between the electrode and dopamine. The changes in currents due to the dopamine electrons released during the oxidation of DA are measured [29,58]. The dopamine oxidation is permanent due to the irreversibility of this reaction under an applied potential; electrons flowing from dopamine to the electrode cannot diffuse out from the electrode surface [59].

### 3.4. Performance of Electrochemical DA Biosensor

**Figure 8:**
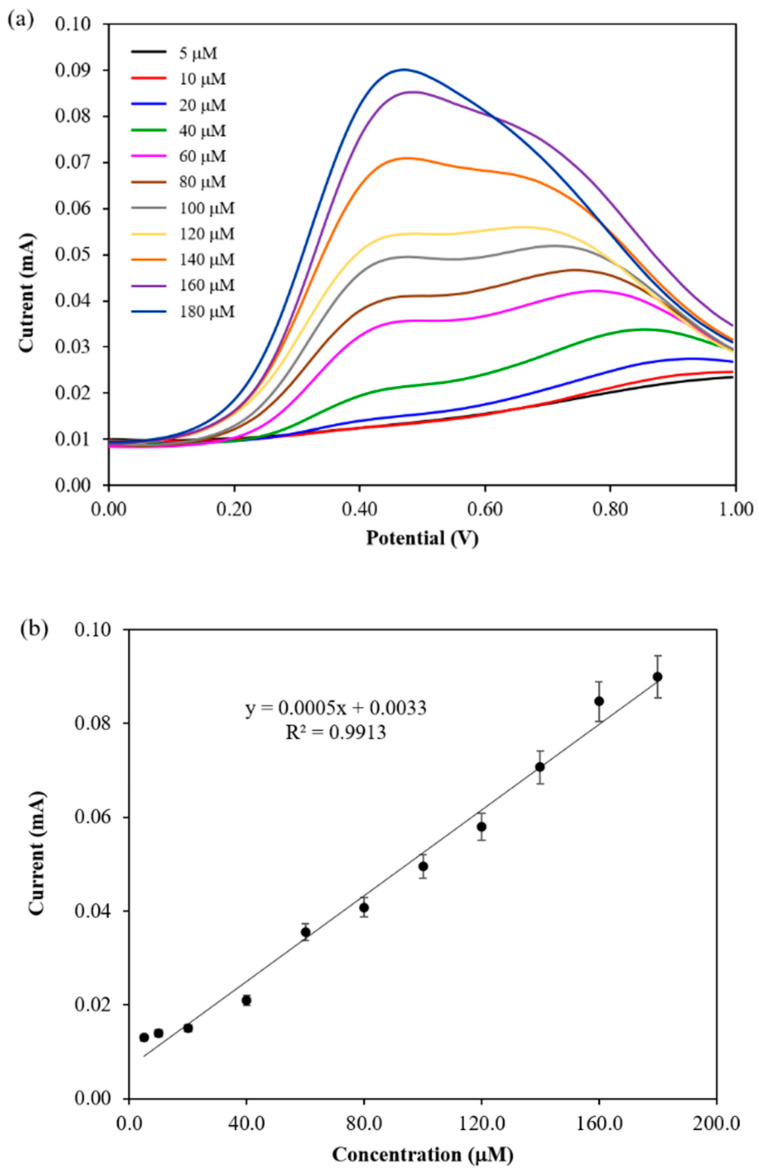
(**a**) DPV responses and (**b**) the calibration curve of PP3C/PPy/AuNPs for 5–180 µM DA.

The electrochemical DA biosensor based on PP3C/PPy/AuNPs was used to detect various concentrations of DA from 5 to 180 µM using the DPV technique. The DPV study demonstrated its precise detection capability with a wide linear range for the DA detection of PP3C/PPy/AuNPs. The DPV responses for various DA concentrations ranging from 5 to 180 µM are illustrated in Figure 8. The oxidation current responses of dopamine were observed at approximately 0.4 V, which gradually increased with increasing DA concentration, as indicated in Figure 8a. A corresponding linear relationship of the current response is shown in Figure 8b. It is expressed in the linear regression equations of I (mA) = 0.0005C_DA_ + 0.0033, with correlation coefficients (r^2^) of 0.9913. The limit of detection (LOD) value was 9.72 nM, and the limit of quantitation (LOQ) value was 0.032 μM, based on the observed linear equation (S/N = 3). The DPV response of PP3C/PPy/AuNPs obtained in PBS solution is presented in Figure S3 of the Supplementary Information. The sensitivity of the electrochemical DA biosensor based on PP3C/PPy/AuNPs was found to be 2 μA μM^−1^ cm^−2^. The DA detection performance of the developed PP3C/PPy/AuNPs-modified electrode was compared with other recent reports as presented in Table 1. The proposed electrochemical biosensor based on PP3C/PPy/AuNPs exhibits comparable or better analytical performance compared to previously reported studies. The primary strength of this study is its broad linear range compared to the method based on the DPV technique. However, the detection limits achieved in this study are less favorable compared to the findings of other research endeavors. Additionally, the fabrication of the biosensor in this study was efficient, involving only a few steps and requiring a short time, which was a significant advantage. Therefore, it could be concluded that the fabricated PP3C/PPy/AuNPs electrochemical biosensor has the potential for detecting DA in real samples.

### 3.5. Determination of Selectivity, Stability, and Reproducibility

**Figure 9:**
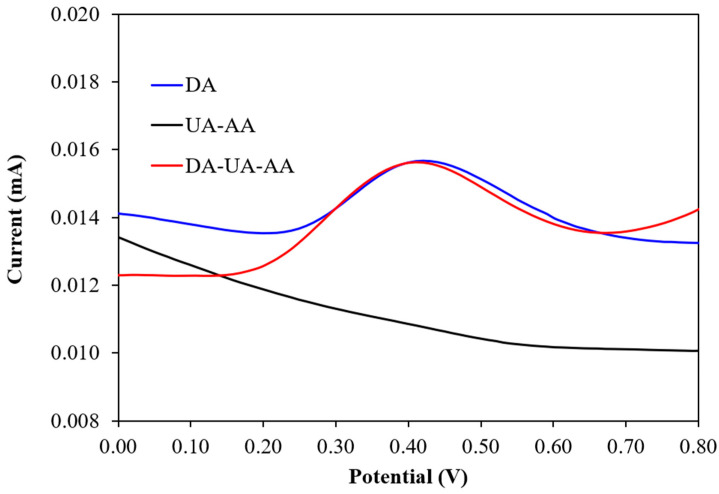
DPV responses of PP3C/PPy/AuNPs for the detection of 20 μM DA, 200 μM AA, and UA, as well as 20 μM DA, with the addition of 200 μM AA and UA interference.

The modified PP3C/PPy/AuNPs electrode for detecting DA was evaluated for selectivity, stability, and reproducibility, which are crucial for its practical applications. The DPV technique was used to evaluate the selectivity of the PP3C/PPy/AuNPs electrochemical biosensor for detecting DA with the addition of AA and UA as interferences. To simulate the coexistence of dopamine bio-molecules, a mixture of 200 μM AA and 200 UA μM was added with 20 μM of DA. The DPV responses of PP3C/PPy/AuNPs to the combination of 20 μM DA with 200 μM AA and UA interferences are shown in Figure 9. The DPV responses demonstrated that the developed sensor exhibits remarkable selectivity for DA detection with negligible current responses observed with the addition of other bio-molecule interferences.

**Figure 10:**
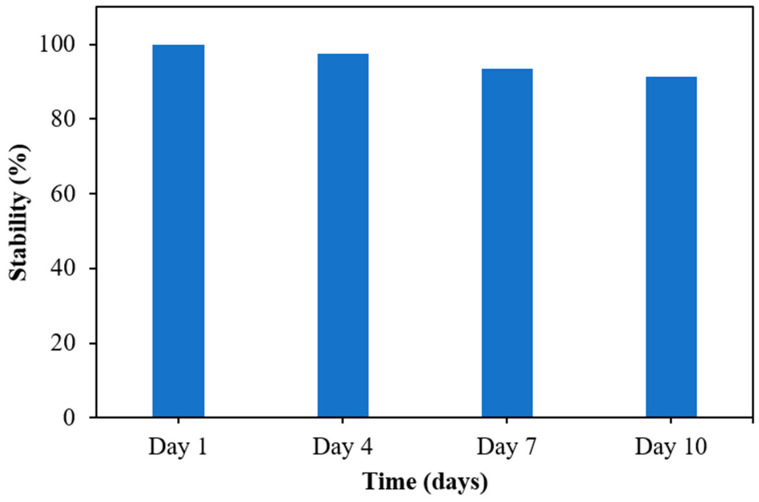
Stability histograms of PP3C/PPy/AuNPs for 100 μM DA.

The stability and reproducibility of the prepared PP3C/PPy/AuNPs electrochemical biosensor were monitored by the DPV technique. To minimize the impact of temperature and humidity on the biosensor’s performance, which can affect binding kinetics, material properties, and electrochemical signals, the biosensor electrodes were stored in a dry environment at room temperature prior to use. The DPV responses, monitored at two-day intervals for 10 days, were analyzed to assess the durability of the biosensor electrodes. As seen in Figure 10, the DPV results indicate that the PP3C/PPy/AuNPs preserved 91.30% of its initial response, highlighting the considerable stability of the prepared PP3C/PPy/AuNPs. Furthermore, the fabrication reproducibility of the prepared PP3C/PPy/AuNPs electrochemical biosensor was investigated. The three electrodes were applied to detect a concentration of 100 μM DA. It was determined that the electrochemical biosensor had a relative standard deviation (RSD) of 4.21%. This result indicates a high level of reproducibility in the fabrication of the PP3C/PPy/AuNPs composite. Thus, it could be concluded that the proposed electrochemical biosensor shows excellent selectivity, high stability, and reproducibility for the detection of DA.

## 4. Conclusions

The PP3C/PPy/AuNPs composite thin film was successfully fabricated by electropolymerization. The presented PP3C/PPy/AuNPs composite thin film exhibited a good electroactivity property in neutral PBS solution (pH 7.4), suggesting potential application for use as an electrochemical DA biosensor. The fabricated PP3C/PPy/AuNPs-modified electrode proved to be sensitive and selective in DA detection through DPV measurements. The fabricated PP3C/PPy/AuNPs exhibited a wide linear detection range, good sensitivity, and high selectivity. The modified electrochemical DA biosensor showed improved efficiency in detecting DA, achieving an LOD of 9.72 nM and a sensitivity of 2 μA μM^−1^ cm^−2^. The obtained results revealed that the electrochemically fabricated PP3C/PPy/AuNPs composite thin film can be applied as a label-free electrochemical biosensor for detecting DA in real samples for the early diagnosis of various neurological disorders in the future.

## Figures and Tables

**Table 1:** Comparison of the PP3C/PPy/AuNPs performance with other electrochemical DA biosensors.

Modified Electrode	Method	Linear Range (μM)	LOD (μM)	Sensitivity	Ref.
PPy–MCM-48	SWV	2–250	0.70	0.0058 (μA μM^−1^)	[19]
PPy/rGO/NiO/GCE	CV	10–1000	0.195	25.89 (A mM^−1^ cm^−2^)	[6]
OONfPPy/PGE	DPV	1–1000	0.00695	-	[60]
ZnO-Cu_x_O-PPy/GCE	DPV	0.1–130	0.04	0.28 (μA μM^−1^)	[61]
PP3C/EOPGE	AdSDPV	0.01–7.5	0.0025	17.23 (μA μM^−1^)	[5]
PP3C/PPy/AuNPs	DPV	5–180	0.00972	2 (μA μM^−1^ cm^−2^)	Present work

Abbreviations: polypyrrole–mesoporous silica molecular (PPy–MCM-48); square-wave voltammetry (SWV); cyclic voltammetry (CV); glassy carbon electrode (GCE); nickel oxide (NiO); polypyrrole (PPy); reduced graphene oxide (rGO), overoxidized nanofiber polypyrrole (OONfPPy); pencil graphite electrode (PGE); differential pulse voltammetry (DPV); zinc oxide (ZnO); copper oxide (Cu_x_O); adsorptive stripping differential pulse voltammetry (AdSDPV); overoxidized pencil graphite electrode (EOPGE); poly(pyrrole-3-carboxylic acid) (PP3C); gold nanoparticles (AuNPs).

## Data Availability

The original contributions presented in this study are included in the article/Supplementary Material. Further inquiries can be directed to the corresponding author.

## Supplementary Materials

The following supporting information can be downloaded at: https://www.mdpi.com/article/10.3390/polym17060754/s1, Figure S1. CV responses of the fabricated PPy and PP3C/PPy/AuNPs composite thin films. Figure S2. The EDX spectrum of PP3C/PPy/AuNPs composite thin film. Figure S3. DPVs of PP3C/PPy/AuNPs obtained in PBS buffer and the presence of 5 μM DA.
