# Supplementary material for: Development of an Electrochemical Biosensor Based on Polypyrrole-3-carboxylic Acid/Polypyrrole/Au Nanoparticle Composites for Detection of Dopamine
Source: Polymers (Basel). 2025 Mar 13;17(6):754. doi: 10.3390/polym17060754 (PMC11944982; doi:10.3390/polym17060754)
Supplement: Supplementary file 1 [file polymers-17-00754-s001.zip › polymers-3478824-supplementary.pdf]

Supplementary Material

Development of an Electrochemical Biosensor Based on Polypyrrole-3-carboxylic Acid/Polypyrrole/Au Nanoparticle Composites for Detection of Dopamine

Rapiphun Janmanee [1,*] and Saengrawee Sriwichai [2,3,*]

1 Chemistry Program, Faculty of Science and Technology, Pibulsongkram Rajabhat University, Phitsanulok 65000, Thailand
2 Department of Chemistry, Faculty of Science, Chiang Mai University, Chiang Mai 50200, Thailand
3 Center of Excellence in Materials Science and Technology, Chiang Mai University, Chiang Mai 50200, Thailand
* Correspondence: rapiphun16@psru.ac.th (R.J.); saengrawee.s@cmu.ac.th (S.S.)

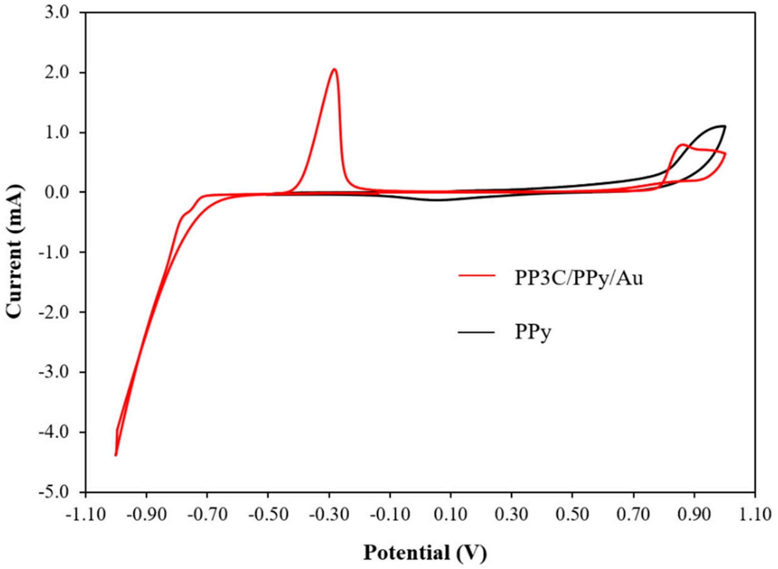

Figure S1. CV responses of the fabricated PPy and PP3C/PPy/AuNPs composite thin films.

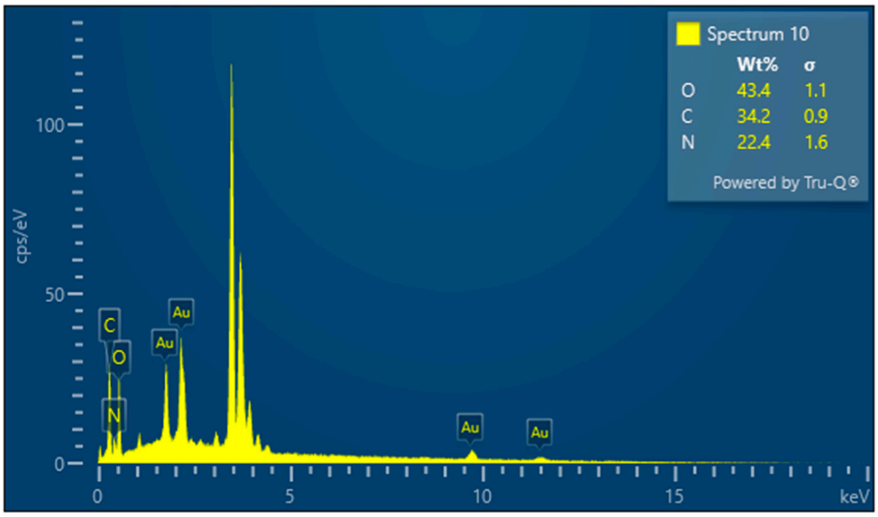

Figure S2. The EDX spectrum of PP3C/PPy/AuNPs composite thin film.

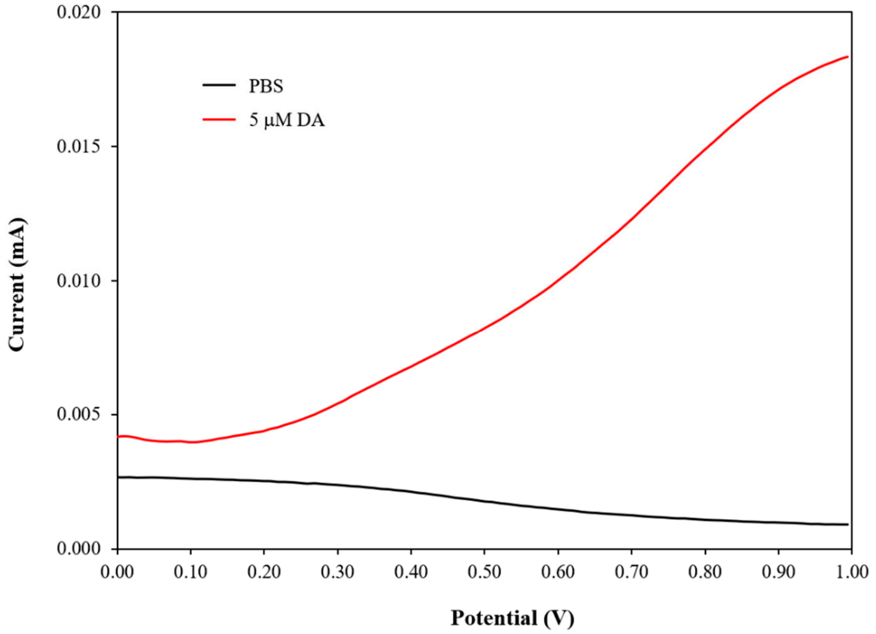

Figure S3. DPVs of PP3C/PPy/AuNPs obtained in PBS buffer and the presence of 5 µM DA.
